# SNRK: a metabolic regulator with multifaceted role in development and disease

**Published:** 2020-08-21

**Authors:** Karthikeyan Thirugnanam, Ramani Ramchandran

**Affiliations:** 1 Department of Pediatrics, Division of Neonatology, Medical College of Wisconsin, Milwaukee, WI 53226, USA.; 2 Obstetrics and Gynecology, Medical College of Wisconsin, Developmental Vascular Biology Program, Children’s Research Institute, Milwaukee, WI 53226, USA.

**Keywords:** Sucrose nonfermenting 1-related kinase, metabolism, inflammation, cardiac function, cardiomyocytes, ischemia, adipocyte, endothelial cells

## Abstract

Sucrose nonfermenting 1-related kinase (SNRK) is a serine/threonine kinase and a member of the adenosine monophosphate (AMP)-activated protein kinase (AMPK) family that is involved in the metabolic regulatory mechanisms in various cell types. SNRK is an important mediator in maintaining cellular metabolic homeostasis. In this review, we discuss the role of SNRK in metabolic tissues where it is expressed, including heart and adipose tissue. We discuss its role in regulating inflammation in these tissues and the pathways associated with regulating inflammation. We also discuss SNRK’s role in vascular development and the processes associated with it. Finally, we review SNRK’s potential as a target in various metabolic dysfunction-associated diseases such as cardiovascular diseases, diabetes, obesity, and cancer. This comprehensive review on SNRK suggests that it has therapeutic value in the suppression of inflammation in cardiac and adipose tissue.

## INTRODUCTION

During embryonic development and cellular differentiation, protein kinases play a vital role in maintaining cellular homeostasis. Protein kinases constitute an exceptionally large family that has been estimated to include more than 1000 mammalian proteins. The sequence similarity of protein kinases in their catalytic domains indicates that they have evolved from a common precursor protein^[1]^. Protein kinases play an important role in signal transduction by phosphorylating specific amino acids of downstream substrates and catalyzing the conversion of substrate proteins into phosphoproteins. The phosphorylation can be reversed by protein phosphatases. Protein phosphorylation is one of the common forms of cellular regulation during various cellular processes including metabolism, proliferation, differentiation, motility, survival, and death. Protein phosphorylation, first described in eukaryotes, is a post-translational modification of proteins whereby a phosphate group is covalently attached to a serine, threonine, or tyrosine residue^[2,3]^. Eukaryotic serine (S), threonine (T), and tyrosine (Y) kinases are grouped together in the eukaryotic protein kinase superfamily based on sequence homology in their kinase domains.

The two main groups of the superfamily, the serine/threonine kinases and the tyrosine kinases can be subdivided further into smaller families which are composed of enzymes that show similar substrate specificities and mode of regulation^[4]^. Serine/threonine kinases (STKs) transfer phosphate group from Adenosine triphosphate (ATP) to the OH (hydroxyl) group on the side chain of a serine or threonine amino acid residue in a protein, producing ADP and a phosphoprotein. STKs are involved in the regulation of cellular proliferation, programmed cell death (apoptosis), cell differentiation, and embryonic development^[5]^. Similar to STKs, tyrosine-kinase enzymes transfer a phosphate group from ATP to a tyrosine residue in a protein. Tyrosine-protein kinases are classified into two main groups: (1) receptor tyrosine kinases, which are attributed to transmembrane proteins involved in signal transduction and play key roles in growth, differentiation, metabolism, adhesion, motility, death, and oncogenesis^[6]^; and (2) cytoplasmic/non-receptor tyrosine kinases, which act as regulatory proteins, playing key roles in cell differentiation, motility, proliferation, and survival^[7]^. Several kinases are activated by auto- or trans-phosphorylation on at least one S/T/Y residue in the activation loop by a second kinase^[8]^.

**Figure 1.**
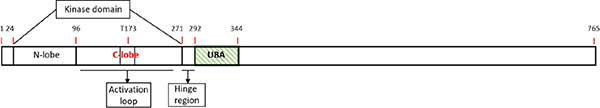
Schematic diagram of SNRK. A linear schematic of the various domains in SNRK is depicted. The numbers on top of the bars denote amino acid. UBA: Ubiquitin-associated domain; SNRK: sucrose nonfermenting 1-related kinase

Sucrose nonfermenting 1-related kinase (SNRK) is a novel member of AMP-activated protein kinase (AMPK) subfamily of STKs. The AMPK family members share sequence homology with other members of the family in their catalytic domain^[4]^. SNRK was first identified in 1996 in 3T3-L1 adipocytes where its expression was observed during differentiation into an adipocyte-like cell^[9]^. SNRK is a monomeric enzyme containing a nuclear localization signal (NLS) domain, an ATP-binding domain, and an active S/T kinase domain with a conserved T-loop threonine residue (T173)^[10]^ [Figure 1]. Kinases that regulate SNRK activity have been identified. For example, liver kinase B1 (LKB1) phosphorylates multiple kinases especially the AMPK family, including SNRK. LKB1 phosphorylates substrates (AMPK and AMPK-related kinases) at the T-loop threonine residue^[11]^. Unlike many AMPKs, SNRK does not require an additional stimulus for activation such as increased AMP:ATP^[12]^ ratio within a cell. SNRK also phosphorylates several proteins including Rho-associated kinase (ROCK)^[12–19]^. Thus, SNRK regulation and its associated signaling partners and pathways are emerging areas of research. Further, SNRK’s role in various cell types in metabolic tissues such as cardiac and adipose is also emerging. We have compiled a list of publications that suggests SNRK’s role in numerous cell types, and its influence on the underlying cellular processes [Table 1]. In this review, we focus on SNRK’s role in the regulation of metabolism and inflammation in heart and adipose tissue, and the impact of SNRK’s dysregulation on metabolic and inflammatory-associated disease conditions.

## SNRK: STRUCTURE, EXPRESSION, AMPK FAMILY ASSOCIATION AND ACTIVATORS

### SNRK structure

The AMPK family members have been extensively characterized. These kinase enzymes include AMPKα1, AMPKα2, MARK1/2/3/4, SIK1/2/3, NUAK1/2, BRSK1/2, and MELK^[25]^. The AMPK protein family contains similar domain organization, namely, an N-terminal kinase domain and an adjacent ubiquitin-associated (UBA) domain. SNRK is a novel member of the AMPK family, and a 2.9Å resolution crystal structure of its N-terminal fragment containing the kinase and adjacent UBA domain is now available^[10]^.

The SNRK sequence is annotated to include a putative kinase domain (residues 24–270) and a hinge region (residues 271–291) which connects to the UBA domain (residues 292–344) [Figure 1]. The kinase domain consists of two lobes namely a N-lobe and a C-lobe. The N-lobe of the kinase domain consists of β-sheets [β2 to β5] and a prominent αC helix. The C-lobe of the kinase domain is mainly α-helical and contains the activation loop^[10]^ [Figure 1]. The UBA domain of the SNRK is composed of three α helices (α1 to α3) and binds to the kinase domain through the hinge region. This binding facilitates interaction of both the N- and C-terminal lobes, which is unique compared to other UBA: kinase domain interactions in the AMPK family. The structure of the UBA domain in SNRK inhibits the kinase activity and thus regulates SNRK’s activity^[10]^. Further, the UBA domain is unique among AMPK family members, and this characteristic triggers and defines specific downstream signals^[26–28]^.

### SNRK activation by upstream kinases

SNRK possesses a conserved threonine (T) residue within its activation loop sequence. However, the identity of the activation loop sequence is not highly conserved among other AMPK-related kinases. LKB1 activates SNRK by phosphorylating its T-residue 173 (T173). The T residue in the activation loop is referred to as “T-loop,” and is part of the three residues Leu (L)-Arg (R)-T that is conserved in the AMPK protein family. An important step in LKB1 activation is its export from nucleus to the cytoplasm, and this nuclear transport of LKB1 requires L-rich peptides. Kinases without a −2 L residue before the T-loop residue cannot get phosphorylated or activated by LKB1 substrate. LKB1 possesses a strong preference to phosphorylate T compared to the L residue at the −2 position^[29]^ in the T-loop. Interestingly, SNRK and the 13 other AMPK subfamily kinases that are phosphorylated and activated by LKB1 (AMPKα1, AMPKα2, MARK1/2/3/4, SIK1/2/3, NUAK1/2, and BRSK1/2) has the L residue at the −2 position in the T-loop. LKB1 gets phosphorylated at S325, T366, and S431 residues by upstream kinases and is auto-phosphorylated at S31, T185, T189, T336, and S404^[30]^ residues. Interestingly, mutation in any of these phosphorylation sites does not significantly affect its intracellular localization^[31,32]^.

*LKB1* was originally identified as a mutated gene in the inherited Peutz-Jeghers Syndrome (PJS), in which subjects are susceptible to developing benign and malignant tumors^[33]^ in the gastrointestinal organs stomach and intestines. LKB1 protein is complexed with STE-related adapter (STRAD), an inactive pseudokinase^[34]^, and mouse protein 25 (MO25)^[35]^, a repeat domain scaffold protein that is responsible for activation of AMPK family kinases. Phosphorylation of S307 residue in LKB1 facilitates the binding of LKB1 to the STRAD and MO25 complex, which enables the nucleocytoplasmic transport of LKB1-complex^[36,37]^ LKB1:STRAD:MO25 complex and Mg-ATP results in a rapid (20 min) 5-fold activation of SNRK in the cytoplasm^[11]^. Therefore, the heterotrimeric complex of LKB1, STRADα or STRADβ, and MO25α or MO25β is required to obtain maximal activation of SNRK.

### Role of SNRK in cardiomyocyte and adipocyte metabolism

**Figure 2.**
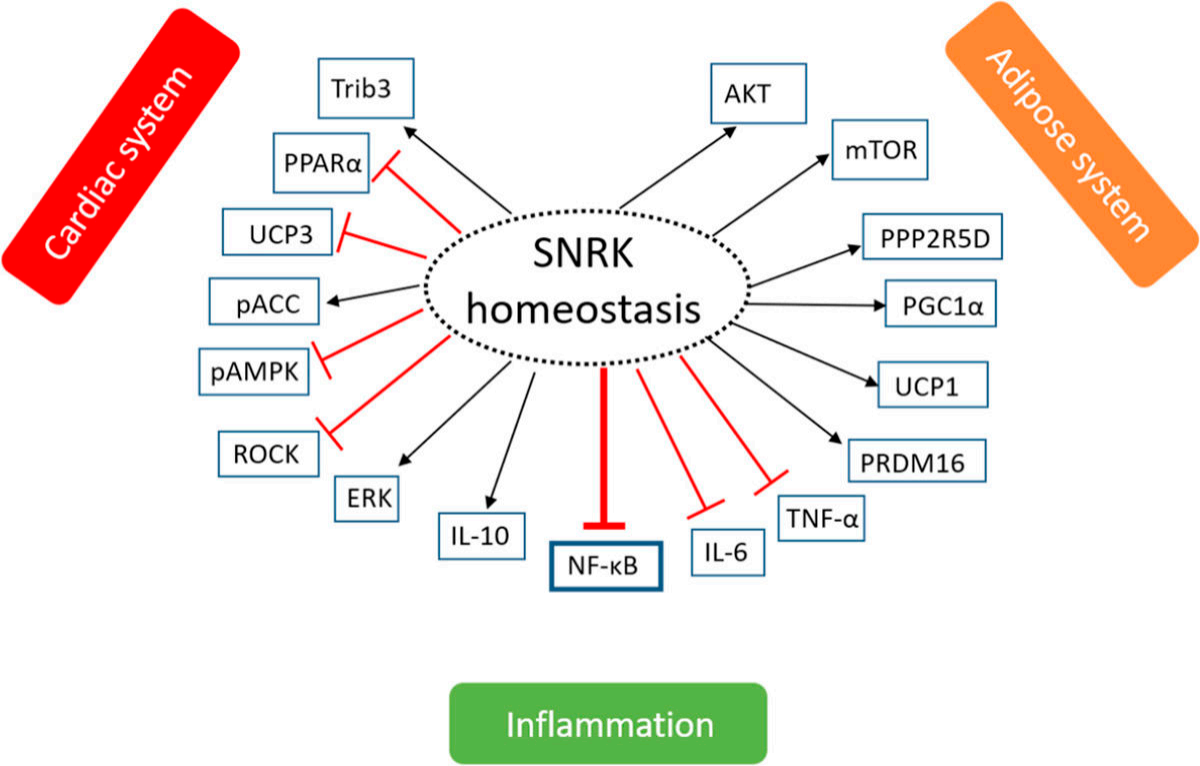
The role of SNRK in maintaining tissue homeostasis. Phosphorylation or signaling of SNRK to maintain homeostasis in three systems - cardiac system, adipose system, and inflammatory system is shown. The arrow indicates whether the phosphorylation is activating (black) or inhibiting (red) for the function of the target protein. The proteins under each system have been implicated to communicate with SNRK in that system. SNRK: sucrose nonfermenting 1-related kinase; Trib3: tribbles homologue 3; PPARα: peroxisome proliferator-activated receptor α; UCP3: uncoupling protein 3; ACC: Acetyl-coA carboxylase; AMPK: adenosine monophosphate-activated protein kinase; ROCK: Rho-associated kinase; ERK: extracellular-signal-regulated kinase; IL-10: interleukin 10; NF-κB: nuclear factor kappa-light-chain-enhancer of activated B cells; IL-6: interleukin 6; TNF-α: tumor necrosis factor α; PRDM16: PR domain containing 16; UCP1: uncoupling protein 1; PGC1α: peroxisome proliferator-activated receptor-γ co-activator 1α; PPP25RD: protein phosphatase 2 regulatory subunit B’Delta; mTOR: mammalian target of rapamycin; AKT: protein kinase B

To facilitate cell growth and maintenance, chemical reactions associated with cellular metabolism such as glucose, fatty acid, and amino acid metabolism occurs within a cell. During periods of stress such as low nutrient environment, the maintenance of cellular metabolism - in turn energy reserves in a cell - is critical. AMPK is a key sensor of energy needs in a cell. SNRK like AMPK is beginning to show similar critical roles in metabolism, and is observed in multiple tissues including adipose and cardiac tissues^[13–19]^. Central to maintaining cellular energy homeostasis is the control of ATP generation and utilization. We discuss next the emerging evidence regarding SNRK’s role in maintaining cellular energy homeostasis [Figure 2].

#### SNRK in cardiac metabolism

In the heart, the myocardium needs to function throughout the life of the organism. In the myocardium, cardiomyocytes (CMs) are the powerhouses that generate energy. In the prenatal or *in utero* heart, glucose and thus glycolysis is necessary for CMs growth. In late gestational and early postnatal stages, glucose uptake is significantly decreased, creating an intracellular glucose deprivation during development^[38]^. In the late phase of cardiac fetal development, circulating lactate contributes to the majority of cardiac oxygen consumption^[39,40]^ while glucose and fatty acid oxidation (FAO) contribute relatively less^[41,42]^. In terms of *Snrk*, during embryonic development, it is expressed at both mRNA and protein levels in tissues that have high demand for metabolic activity^[19]^. The heart is comprised of vascular endothelial cells (ECs) and smooth muscle cells, in addition to CMs, all of which express SNRK^[15]^. Loss of *Snrk* in all tissues [global knockout (KO)] results in neonatal lethality with enlarged hearts observed at E17.5 and P0^[15]^. Microarray analysis of E17.5 hearts revealed systemic metabolic dysregulation^[15]^. Glycogen, a polysaccharide (serves as glucose storage) was decreased in E17.5 *Snrk* global KO hearts. Similarly, lipid storage deposits demonstrated by oil red O (ORO) staining was also less in E17.5 *Snrk* global KO heart tissue. Circulating lipid plasma levels were also lower in *Snrk* global KO mice. Thus, global loss of *Snrk* results in defects associated with cardiac tissue energy sources.

The phospho-AMPK-phospho-acetyl-CoA carboxylase (ACC)^[15]^ is one of the key signaling pathways associated with cardiac metabolism in neonates and adults. AMPK decreases FAO by phosphorylation of ACC1 at S79 and ACC2 at S212 residue. ACC enzymes generates malonyl CoA from acetyl CoA (a byproduct of FAO pathway). Phosphorylation of ACC is inhibitory and thus prevents the generation of malonyl CoA. This relieves the inhibition on a transporter (carnitine palmitoyltransferase) that allows acyl CoA to enter mitochondria for b-oxidation and tricarboxylic acid (TCA) cycle to generate acetyl CoA and ATP respectively. In P0 *Snrk* global KO hearts, pAMPK and pACC levels were down compared to wild type hearts suggesting malonyl CoA accumulation and thereby inhibition of carnitine palmitoyltransferase and loss of b-oxidation or FAO^[15]^. Thus, SNRK promotes FAO via the pAMPK-pACC pathway in the neonate P0 hearts.

**Figure 3.**
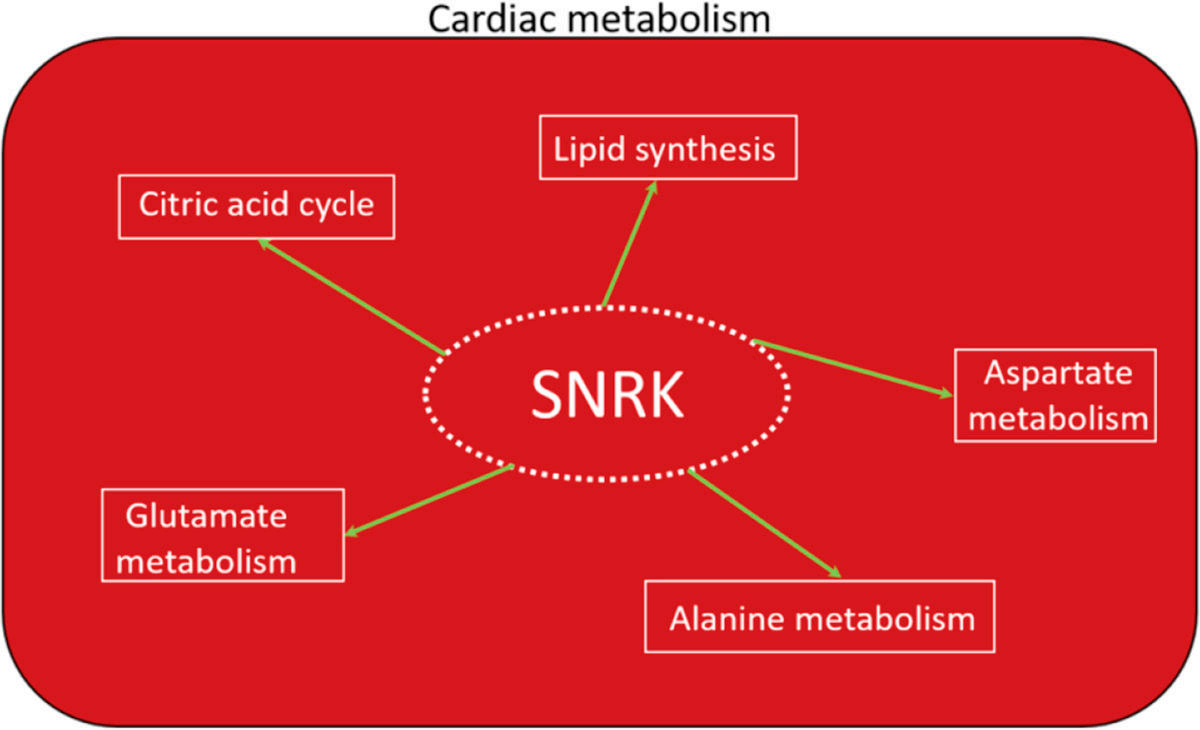
SNRK in cardiac metabolism. The role of SNRK in various metabolic pathways involved in cardiac functioning is depicted. SNRK: sucrose nonfermenting 1-related kinase

As the transition from the neonate to postnatal stage ensues, the heart of a newborn will adapt to the changing environment which is characterized by an increase in contractile demand due to the rapid growth and increase in activity of the newborn^[43]^. This results in an increase in energy demand that can be provided by FAO and mitochondrial ATP production. The high fatty acid content of the maternal milk in many species is an effective way to supply the high energy demand of the newborn heart. As the newborn heart matures, the utilization of FAO to meet the overall energy production needs increases, and thus becomes the dominant substrate in the adult heart. Under normal metabolic conditions, over 95% of ATP generated in the heart is derived from oxidative phosphorylation. Only 5% of the remaining comes from glycolysis and to a lesser extent from the TCA cycle^[44]^. Accordingly, the high-energy phosphate pool in the heart, ATP, is relatively small and can be exhausted within a few seconds. Therefore, cardiac work depends strongly on ATP generation, and impairments in this process can rapidly induce contractile dysfunction. Of the ATP generated in the adult heart, 70% to 90% is produced by the oxidation of fatty acids (or FAO). The remaining 10% to 30% comes from the oxidation of glucose and lactate, as well as small amounts of ketone bodies and certain amino acids^[45,46]^. Because *Snrk* global KO mice die at or before birth, studying SNRK function in adult requires conditional deletion of SNRK in cardiac tissue using the CRE-LoxP system. Cardiomyocyte-specific (myh6-CRE) *Snrk* KO mice (*Snrk* cmcKO) show cardiac functional deficits at 6 months and die at 9 months. No neonatal lethality was observed in these mice^[15]^. Neonate hearts from the *Snrk* cmcKO mice showed higher ORO retention and no change in pACC-pAMPK signaling pathway. However, adult *Snrk* cmcKO hearts at 6 months showed no change in ORO but showed higher pACC (unpublished data) levels. Thus, the switch in energy source for the heart from glucose in neonates to fatty acids in adults may partly reflect SNRK’s role at these time points. In terms of cell type, where SNRK function is critical in the heart, there is little doubt that SNRK-CM function is dominant. This is supported by the following evidence: (1) *Snrk* cmcKO show profound cardiac functional deficits at 6 months, die at 9 months, and when stressed by Angiotensin II (Ang II) at 4 months, they show cardiac function deficits within 14 days and die; (2) *Snrk* endothelial (TIE2) cell conditional KO (*Snrk* ecKO) do not show cardiac functional deficits at 6 months, are alive, and when stressed with Ang II at 4 months do not show cardiac functional deficits; and (3) *SNRK* knockdown cardiomyocytes *in vitro* show metabolic deficits, and NMR (nuclear magnetic resonance)-based metabolomic analysis revealed SNRK is essential for alanine, aspartate, and glutamate metabolism [Figure 3] as well as TCA cycle metabolism and it also regulates metabolites involved in lipid synthesis such as glycerol^[47]^. It is also noteworthy that pAMPK-pACC levels were significantly altered in *Snrk* ecKO neonate hearts and also in adult hearts (unpublished data). But, despite these alterations, the SNRK in CMs seem to compensate for cardiac function, and thus prevents functional deficits.

As for the targets of SNRK that are involved in cardiac function, we had previously reported that ROCK was a putative SNRK substrate in CMs [Figure 2], and showed that Fasudil (ROCK inhibitor) can rescue cardiac functional deficits in *Snrk* cmcKO hearts^[13]^. ROCK signaling pathway^[13]^ activation is also implicated in major cardiovascular disorders such as atherosclerosis, restenosis, hypertension, pulmonary hypertension, and cardiac hypertrophy^[48]^. Tribbles homologue 3 (Trib3) is another substrate of SNRK in the heart [Figure 2], and SNRK overexpression in the heart decreases oxygen consumption and improves cardiac function^[16]^. Trib3 is also a known inhibitor of AKT signaling^[49]^ and metabolic flux is maintained by PPARα-dependent UCP3 (uncoupling protein 3) downregulation^[16]^. Thus, SNRK improves cardiac mitochondrial efficiency and decreases mitochondrial uncoupling^[16]^. Collectively, SNRK acts as a cardiomyocyte-centric metabolic sensor in cardiac tissues to maintain cardiac function and homeostasis, and is a novel candidate to target in order to improve cardiac health.

#### SNRK in adipocyte metabolism

Adipose tissue is a loose connective tissue composed of adipocytes, cells which contain either a single large lipid droplet (white adipose tissue) or multiple lipid droplets (brown adipose tissue). Adipocytes release fatty acids into the bloodstream via lipolysis of lipids. Adipose is a highly dynamic tissue and contains adipocytes of various size referred to as small and large adipocytes. The properties of adipocytes cells have been extensively explored for the relationship between cell size and various disease conditions such as inflammation^[50,51]^, insulin resistance^[52,53]^, and diabetes^[54,55]^. The correlation between its size and cellular function as well as in metabolic disease concluded that the size of the adipocyte is an important factor in predicting pathophysiological conditions^[56]^.

The most known and recognized function of adipose is its role in the storage and release of lipid species, particularly free fatty acids^[57]^. SNRK is ubiquitously and abundantly expressed in both white adipose tissue (WAT) and brown adipose tissue (BAT)^[19]^. Phosphoproteomic analysis revealed *SNRK* knockdown in adipocytes significantly decreased phosphorylation of 49 proteins by 25% or more and increased phosphorylation of 43 proteins by onefold or higher. Among these proteins, several were involved in the inflammatory pathways. Pathways such as mTOR signaling were implicated in addition to those that reduce adipocyte function^[19]^. In adipocytes, acute inhibition of mTOR signaling by rapamycin increases insulin-stimulated glucose uptake, but chronic inhibition of mTOR signaling (rapamycin) impairs insulin-stimulated glucose uptake^[58]^. Thus, SNRK’s role in regulating insulin-mediated glucose uptake is context-dependent. In support of this hypothesis, a recent study, from Li *et al.*^[17]^, reported that SNRK controls insulin signaling through Protein Phosphatase 2 Regulatory Subunit B’Delta (PPP2R5D) phosphorylation [Figure 2], which subsequently influences protein phosphatase 2A (PP2A) activity and phosphorylates AKT in both WAT and BAT. PPP2R5D is one of the four major Ser/Thr phosphatases implicated in the negative control of cell growth and division. This implies that SNRK activates insulin-stimulated AKT phosphorylation and glucose uptake in adipocytes. Further, SNRK, specifically in adipocytes, maintains body weight but it does not change the size of the WAT depot^[18]^. SNRK keeps circulatory triglycerides and free fatty acids in check in order to regulate the body weight. These data imply that SNRK is a potential target for interference in adipocytes to check body weight.

#### SNRK and inflammation

Tissue inflammation is a key protective mechanism to promote repair caused by ischemia and to prevent further damage. SNRK appears to control tissue inflammation especially by suppressing the inflammatory pathways mediated through nuclear factor kappa-light-chain-enhancer of activated B cells (NF-κB) signaling^[14,22]^ [Figure 2]. To restore normal tissue architecture, post-injury inflammation occurs in three distinct phases. In the early proinflammatory first phase, components of the innate immune response initiate the repair by mobilizing the recruitment of key inflammatory cells. In the second phase, the proinflammatory response begins to diminish and inflammatory cells such as macrophages switch phenotype to a reparative mode. In the final phase, tissue homeostasis is reestablished when the inflammatory cells either withdraw from the site of injury or are abolished through apoptosis. However, the degree and duration of the response varies, and this dictates whether the final outcome of the inflammation will be beneficial or harmful. A prolonged inflammatory response has negative consequences such as activation of a fibrotic response where excessive and aberrant accumulation of collagenous connective tissues occurs in the organs that can weaken tissue function, and in some cases, lead to organ failure (e.g., chronic hepatitis B)^[22,59,60]^.

Often, inflammation is associated with fibrosis in cardiac, adipose and renal tissues^[8,14,22,61]^. Evidence in recent years point to a strong link between chronic low-grade inflammation in the heart and metabolic dysregulation^[62–64]^. In the case of heart failure and cardiac hypertrophy, the progression of inflammation usually involves a local rise of cytokines in cardiac cells such as CMs, ECs, and fibroblasts and the activation of the proinflammatory transcription factor nuclear factor NF-κB^[14,65,66]^. In the cardiac system, inflammation is often associated with the deposition of collagen, leading to fibrosis in the heart. Removal of *Snrk* in CMs increases the phosphorylation of NF-κB p65 and increases proinflammatory cytokine signaling which is partially mediated through Akt. This suggests that SNRK represses inflammation signaling in CMs, which when unchecked, progresses to fibrosis and death. However, when *Snrk* is deleted in cardiac ECs, these hearts show increases in NF-κB p65 and proinflammatory cytokine signaling, which does not progress to fibrosis. These data collectively suggest that SNRK in CMs compensates for SNRK loss of function in ECs^[14]^, and also implies crosstalk signaling between CMs and ECs in cardiomyocyte remodeling.

Healthy adipose tissue is vital for metabolic homeostasis, whereas dysfunctional adipose is a contributing factor for metabolic disorders such as obesity, type 2 diabetes mellitus, and cardiovascular diseases^[67–69]^. SNRK expression is high in the metabolic adipose tissue. In the adipocytes, SNRK protein is localized to lysosomes, the site for degradation of large intracellular organelles or assembly of protein aggregates^[19]^. Adipocyte-specific SNRK represses inflammation in WAT through the JNK and IKKβ signaling pathways. Under obesity conditions, the expression level of SNRK is low because of obesity-induced adipose inflammation and/or lipid toxicity. Removal of SNRK specifically in adipocytes leads to metabolic disturbances with decreased energy expenditure, higher body weight, and increased insulin resistance^[18,19]^, suggesting that SNRK regulates and controls these metabolic pathways.

In the renal system, SNRK in glomerular ECs binds directly to p65 subunit of NF-κB (activated by Ang II) to suppress inflammatory signaling. Activated NF-κB plays a critical role in renal damage which occurs as a result of unchecked inflammation and fibrosis^[22]^. These evidences suggest that SNRK acts as a repressor of inflammation in cardiac, adipose, and renal tissues, all of which are critical for the maintenance of organismal homeostasis and function.

### Role of SNRK in vascular development

During embryonic development, vascular networks permeate the entire body and this conduit is used for the circulation of metabolites and removal of waste products. The vascular system emerges as one of the earliest networks in the embryo to support the rapid growth of tissues. The adult vasculature is generally quiescent but retains the capacity to shift from this dormant state to an expansion and remodeling state. This occurs during normal physiological conditions such as wound healing or pathological states such as tumor neovascularization. The differentiation of angioblasts (precursor cells) into ECs and the *de novo* formation of a primitive vascular network is called vasculogenesis. After primary vascular plexus is formed, a second process during development occurs wherein more ECs are generated via a process called angiogenesis which is the growth of new blood vessels from existing vasculature^[70]^. Thus, vasculogenesis and angiogenesis are the two predominant sequential coordinated processes for blood vessel formation during development^[70,71]^.

#### SNRK in vasculogenesis

The initial loss of function studies *in vivo* for *Snrk* was performed in the vertebrate zebrafish model system. Zebrafish offers various advantages as a model system including established genetics, loss and gain of function technology, transparency of embryos, fluorescent transgenic reporter lines, *ex vivo* development of embryos, and several others^[72–74]^. During embryonic development, angioblasts migrate from lateral plate mesoderm to the midline, differentiate in arterial or venous endothelial cells and coalesce to form cord-like structures which eventually mature to form the major axial blood vessels, namely dorsal aorta (DA) and posterior cardinal vein (PCV)^[75]^. The kinase function of SNRK was shown to be essential for angioblast migration and is required for localization and maintenance of these cells in the lateral plate mesoderm^[76]^. SNRK also functions later (19–22 h post-fertilization) during angioblast differentiation to arteries and veins in zebrafish where it is involved with Notch signaling-mediated arterial or venous (A/V) specification. Studies in zebrafish reveals that within 2 h of the formation of angioblasts, angioblasts begin their migration to the dorsal midline, and by 18 h post-fertilization, angioblasts coalesce at the midline to form the two major axial vessels (DA & PCV)^[77,78]^. Thus, sufficient number of angioblasts are needed in the embryo to facilitate this process. As described earlier, most kinases are counter balanced by phosphatases. In the lateral plate mesoderm, dual-specific phosphatase 5 (DUSP5), a member of MAPK phosphatases was identified as a vascular-specific gene in 2 independent microarray studies^[74,79]^. Subsequent analysis shows that Dusp-5 phosphatase and SNRK kinase function together in maintaining angioblast populations during embryonic vascular development^[20]^. Thus, the signaling pathway and the targets that are regulated by SNRK and DUSP5 are likely to reveal more understanding of the basic vasculogenesis process during vertebrate organogenesis.

#### SNRK in angiogenesis

The main mediator of angiogenesis is the arrangement of ECs in tip and stalk cells^[80]^. Tip cells containing filopodia invade surrounding tissue leading to the path of neo-vessel formation. Vascular endothelial growth factor and Notch signaling pathways are vital for tip cell differentiation^[80]^. Most of our current knowledge about the morphological processes and molecular regulation of angiogenesis are from the developing zebrafish embryos or the vascularization of postnatal mouse retina^[80,81]^. In zebrafish, the accessibility of embryos for live imaging provided unprecedented observations which have unraveled new concepts in angiogenesis. Analysis of the zebrafish head and trunk vasculature reveals that SNRK controls patterning of vessels in both locations^[20]^. In retina, the segregation of vascular and avascular compartments and the visualization of the progressive expansion of blood vessels from the center to periphery makes it an attractive model to study tip versus stalk cell formation during angiogenesis. In this model, endothelial SNRK was found to be essential for vascular patterning in that it controls vessel diameter and vascularization area in the retina^[21]^. This regulation is partially mediated through transcription factor hypoxia-inducible factor-1a (HIF1α), which is induced under a physiological drop in oxygen concentration below 60 mmHg, a condition referred to as hypoxia. The hypoxia state in tissue often induces angiogenesis. Similarly, during both acute and chronic myocardial ischemia, angiogenesis is stimulated. Ischemia-driven angiogenesis is primarily an adaptive physiological response to either an increase in tissue mass or elevated oxygen consumption^[82]^. Under ischemic condition, HIF1α is upregulated and was shown to interact directly to *SNRK* promoter^[21]^. This binding results in transcriptional upregulation of *SNRK* mRNA, which then promotes angiogenesis by activating ITGB1 (β1 integrin)-mediated EC migration^[21]^. Thus, SNRK plays a vital function in developing vasculogenesis and ischemic angiogenesis. However, its exact role and the underlying mechanisms associated with facilitating normal angiogenesis remains unknown.

### Role of SNRK in disease

Inflammation and metabolic dysregulation are major contributing factors to disease. Because SNRK participates in both processes, it is considered an important target for intervention. Based on SNRK’s preponderance as a repressor of cellular functions, we consider SNRK as a checkpoint in cells. Thus, therapeutic approaches that support SNRK agonist development is preferred. Further, SNRK’s role at the interface of inflammation and metabolism will benefit conditions such as heart failure or diabetes. We hypothesize that molecules such as SNRK that work at interfaces of inflammation-metabolism will help reduce the progression of the disease. In addition, SNRK’s ability to suppress inflammation in multiple systems, such as cardiac, adipose, and renal, opens avenues for therapeutic development in these organ systems.

#### SNRK in heart failure

**Figure 4.**
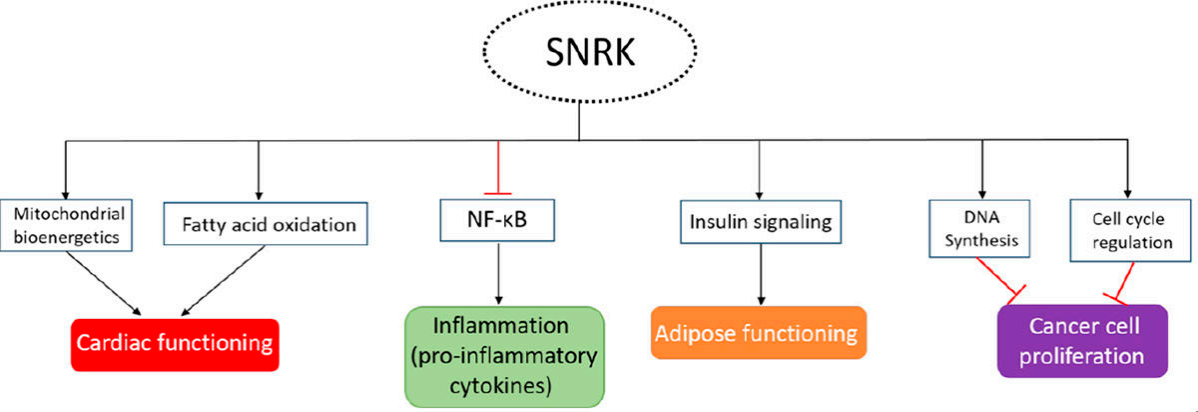
Important functions of SNRK. The important function of SNRK in various signaling paradigms, and consequence associated with that in respective tissues is depicted. The arrows indicate activation (black) and inhibition (red). SNRK: sucrose nonfermenting 1-related kinase

Progressive heart failure (HF) is a chronic condition, and current therapies targeting HF are insufficient. HF is a leading cause of morbidity and is associated with increasing mortality rate worldwide. HF is often accompanied with significant perturbations in energy metabolism that can affect both cardiac energy supply and efficiency^[83]^. HF is also associated with several underlying comorbidities including dilated cardiomyopathy, myocardial infarction, hypertension, and myocarditis. Metabolic changes or dysfunctions in cardiac tissue are one of the main reasons for HF progression. Prolonged exposure of metabolic stress in the heart decreases its functional ability, especially by reducing mitochondrial function, which is the main energy generating source in the heart. Mitochondrial dysfunction appears to be a vital target for direct intervention to improve cardiac function because it primarily uses fatty acids for ATP production^[84]^. SNRK is involved in the regulation of the mitochondrial substrate usage and oxygen consumption to maintain cardiac energy and functioning^[16]^. Progressive HF reduces free fatty acid breakdown resulting in less ATP production, more inflammation, and increased fibrosis. SNRK in CMs regulates cardiac energy homeostasis by maintaining FAO^[15]^ [Figure 4]. Loss of functionally active SNRK in CMs makes the heart vulnerable and the mice succumb in approximately 9 months. Any additional stress such as angiotensin II (Ang II) accelerates HF and the mice dies in two weeks post Ang II infusion. Cardiac functional parameters are severely compromised with upregulation of inflammation and fibrosis markers^[14]^ in the heart tissue. HF is typically associated with cardiac remodeling where inflammation and fibrosis are thought to play crucial roles^[60]^. These studies suggest that maintaining SNRK function in CMs is key to preventing HF. In the heart, in addition to CMs, ECs are also present in higher numbers than previously thought^[85]^. Interestingly, when SNRK was deleted in ECs, the hearts from these mice did not show cardiac function deficits in wild type state or in Ang II-induced state. As mentioned earlier, the NF-κB pathway was activated in these hearts [Figure 4], but no fibrosis was observed^[14]^. These studies suggest an overriding role for SNRK in CMs and its compensation of functional defects elicited by other cell types in the heart. Further, SNRK in CM keeps inflammation and fibrosis under check which allows the heart to continue its function. These studies suggest important concepts that require more investigation as it relates to SNRK’s role in HF, which include: (1) SNRK-mediated cell-cell communication in the heart; (2) pharmacological activation of SNRK selectively in CMs to suppress inflammation and metabolic dysregulation; and (3) SNRK activation in CMs to promote cardiac output via mitochondrial or other mechanisms. Some of SNRK’s cardiac function are reminiscent of mitochondrial sirtuin proteins specifically SIRT3, wherein *Sirt3* knockout mice are highly sensitive to stress, which leads to cardiac hypertrophy, fibrosis, and increased mortality^[86]^. Compounds such as Honokiol that activate mitochondrial Sirt3, block and reverse cardiac hypertrophy in mice^[87]^ and show protective cardiac function^[88]^ could be candidates for testing in *Snrk* cmcKO mice. Collectively, the therapeutic value of SNRK-CMs-mediated signaling to prevent HF is an emerging area of translational research in cardiovascular medicine.

#### SNRK in diabetes

Diabetes mellitus is often referred to as a metabolic condition that results in high blood glucose levels. Type 1 diabetes (T1D) is a severe form of the disease and is often referred to as juvenile diabetes or “insulin-dependent diabetes” which is the result of loss of insulin-hormone producing islet cells in the pancreas which normally promotes glucose metabolism. Type 2 diabetes (T2D), the most common form of diabetes, is also referred to as adult onset diabetes or “non-insulin-dependent diabetes.” In T2D, the insulin receptor is defective and insulin produced by the pancreatic cells cannot function to facilitate efficient glucose metabolism. This is often referred to as “insulin-resistance” state. Insulin secreted by pancreas influences other organs including muscle (glucose uptake and storage), liver (decrease glucose production), and adipocytes (increased lipogenesis). SNRK’s connection to diabetes was first identified by studying its role on adipocytes^[19]^. SNRK is abundantly expressed in adipose tissue (WAT and BAT), and its expression is induced by insulin. *SNRK* knockdown in adipocytes promotes lipolysis, impairs glucose uptake^[17]^, and activates NF-κB inflammatory signaling pathway. Thus, SNRK, like in CMs, seems to function in adipocyte as a repressor of adipocyte inflammation. The specific mechanism utilized by SNRK to prevent insulin resistance in adipose tissue is through protein phosphatase 2 regulatory subunit B’ delta (PPP2R5D) phosphorylation, which impacts PP2A activity and phosphorylation of AKT^[17]^ [Figure 4]. The SNRK-AKT connection is intriguing given that this signaling nexus was also observed in heart tissues from *Snrk* cmcKO mice^[14]^. Thus, further investigations is needed into the direct versus indirect regulation of SNRK-AKT pathway in CMs and adipocytes.

Obesity is a key risk factor for insulin resistance T2D^[89]^. Interestingly, in humans and mouse models of obesity, adipose SNRK expression levels are diminished. Further, adipocyte-specific deletion of *Snrk* causes inflammation in WAT along with ectopic lipid deposition in liver and muscle^[18]^. Homozygous loss of *Snrk* in adipocytes decreases the expression of uncoupling protein 1 (UCP1), PR domain containing 16 (PRDM16), and Peroxisome proliferator-activated receptor gamma coactivator 1-alpha (PGC-1α) in BAT. One of the primary functions of adipocytes is to insulate the body, a process also referred to as “thermogenesis”. All three molecules UCP1, PRDM16, and PGC-1α play an important role in BAT thermogenesis^[18]^. To increase heat production, lipids in adipocytes are catabolized, a process that gets dysregulated in obese conditions, which results in increased inflammation and insulin resistance. Adipocyte-specific deletion of *Snrk* cause impairment in adaptive thermogenesis in BAT leading to decreased energy expenditure, elevated body weight, and insulin resistance. Importantly, a significant association in *SNRK* genetic variants and obesity risk was identified in humans^[18]^. These studies collectively make a case for SNRK as a novel target for treating obesity and insulin resistance-related metabolic disorders including diabetes.

#### SNRK in cancer

Reprogramming of cellular energy metabolism is one of the principal hallmarks of cancer^[90]^. We think that tumor cells can exploit SNRK’s role in controlling metabolic pathways in various tissues. For example, LKB1, an upstream regulator of SNRK function, has been identified as a critical cancer suppressor protein and is mutated in several types of cancers^[91–96]^. LKB1 may inhibit cancer cell growth through regulation of HIF-1 under hypoxic condition. Hypoxia is an important characteristic in most cancers, and induces the expression of HIF-1 transcription factor. HIF-1 can subsequently activate genes that permit cancer cells to survive and grow in the hypoxic tumor environment^[97]^. HIF1α binds to the *SNRK* promotor during ischemia, and induces it expression. SNRK protein is found in both cytoplasm and in the nucleus and regulates genes involved in DNA synthesis and cell cycle regulation [Figure 4]. Overexpression of *Snrk* decreases cell proliferation, whereas downregulation of *Snrk* increased cell proliferation in colon cancer cell lines. Mechanistically, SNRK inhibits the proliferation of colon cancer cells through upregulation of calcyclin-binding protein (CacyBP) and β-catenin degradation^[12]^. CacyBP is a tumor suppressor which has been implicated in reducing cancer cell proliferation through regulating cell cycle G1 check point in breast^[98]^, gastric^[99]^, and kidney cancers^[100]^.

Another tumor type that depends extensively on host metabolism are ovarian cancer cells^[23]^. Adipocytes in the omentum (fat layer underlying the belly) microenvironment^[101]^ provide fatty acids as source of energy to ovarian cancer cells to support their rapid growth, progression, and metastasis^[24]^. In ovarian cancer, the expression of SNRK is lower in metastatic tumors and is differentially expressed depending on the stage of the disease. This suggests that SNRK has specific roles in the disease progression of ovarian cancer^[98]^ and may have diagnostic value for stratifying ovarian tumors of varying types. However, much work is needed to realize SNRK’s full potential and its importance in cancer biology.

In summary, therapeutic strategies directed towards the enhancement of SNRK function could significantly improve the clinical conditions associated with inflammation and metabolic dysfunction.

## CONCLUDING REMARKS AND FUTURE PERSPECTIVES

A considerable amount of evidence supports the notion that SNRK activation may act as a suppressor of inflammation and metabolic processes. Inflammation suppression comes with direct inhibition of NF-κB-mediated inflammatory signaling, one of the signature pathways that SNRK controls. SNRK’s role in regulating glucose and fatty acid metabolism is considered significant for the function of cardiac and adipose tissues. Given that mitochondria is a major source of ATP production in the cell, it is not surprising that SNRK is involved in regulating mitochondrial bioenergetic potential in CMs. Emerging evidence in SNRK biology also suggests that it plays a defining role in cell-cell communications in various tissues that will extend beyond adipose, renal, and cardiac tissues. In adipose tissues, SNRK plays a predominant anti-inflammatory role. In renal tissues, endothelial SNRK protect the kidney epithelium from becoming fibrotic. In the cardiac tissues, CMs SNRK is necessary for protecting ECs from undergoing fibrosis. In addition, SNRK role in phosphorylating proteins (substrates) whether directly or indirectly to either promote or inhibit its target activity/signaling pathway will need extensive evaluation and is the future of SNRK signaling. Finally, SNRK’s role in causative human disease phenotypes will cement the importance of this molecule from a translational biology perspective. The next 10 years of SNRK biology will be interesting and exciting to witness, and our group along with many others will benefit from this knowledge which we hope one day will impact patients’ lives in the form of SNRK-centric novel treatments.

## Figures and Tables

**Table 1.** The role of SNRK in various cellular systems is shown

System	Function	Role of SNRK in the system	Ref.
Cardiac system	Cardiac metabolism	Regulates cardiac metabolism through phospho-acetyl-CoA carboxylase (ACC) and phospho-AMPK signaling pathway	[15]
	Cardiac functioning	Regulates Rho-associated kinase (ROCK) signaling pathway and mitochondrial efficiency through uncoupling protein 3 (UCP3) and mitochondrial uncoupling	[13,16]
	Cardiac inflammation	Represses inflammation by regulates NF-κB phosphorylation	[14]
Adipose system	Adipocyte glucose metabolism	Regulates insulin signaling mediated glucose uptake through PPP2R5D and Akt phosphorylation	[17]
	Adipocyte inflammation	Represses inflammation in white adipose tissue through JNK and IKKβ pathways	[19]
	Adipose thermogenesis	Represses WAT inflammation and regulate BAT thermogenesis through UCP1 and PGC1α	[18]
Vascular system	Vasculogenesis	Maintain angioblast populations and control angioblast numbers in embryonic vascular development through DUSP5	[20]
	Angiogenesis	Promote endothelial angiogenesis by activating ITGB1 (β1 integrin)-mediated endothelial cell migration	[21]
Renal system	Kidney inflammation	Represses inflammation by directly interacting with NF-κB phosphorylation	[22]
Colorectal system	Colon cancer	Inhibits colon cancer cell proliferation through upregulation of calcyclin-binding protein (CacyBP) and β-catenin degradation	[12]
Ovarian system	Ovarian cancer	Omental adipocytes transport fatty acids for rapid growth, progression, and metastasis of ovarian cancer cells	[23]
Neuronal system	Neuron apoptosis	Regulates low K^+^- induced apoptosis in cerebral neurons	[24]

SNRK: sucrose nonfermenting 1-related kinase; AMPK: AMP-activated protein kinase; NF-κB: nuclear factor kappa-light-chain-enhancer of activated B cells; PPP2R5D: serine/threonine-protein phosphatase 2A 56 kDa regulatory subunit delta isoform; Akt: protein kinase-B; JNK: Jun N-terminal kinase; IKKβ: IκB kinase β subunit; WAT: white adipose tissue; BAT: brown adipose tissue; PGC1α: peroxisome proliferator-activated receptor γ isoform α; DUSP5: dual-specificity phosphatase 5; ITGB1: Integrin beta-1
